# *Bifidobacterium* β-Glucosidase Activity and Fermentation of Dietary Plant Glucosides Is Species and Strain Specific

**DOI:** 10.3390/microorganisms8060839

**Published:** 2020-06-03

**Authors:** Nikol Modrackova, Eva Vlkova, Vaclav Tejnecky, Clarissa Schwab, Vera Neuzil-Bunesova

**Affiliations:** 1 Department of Microbiology, Nutrition and Dietetics, Czech University of Life Sciences Prague, Kamycka 129, 16500 Prague 6, Czech Republic; modrackova@af.czu.cz (N.M.); vlkova@af.czu.cz (E.V.); schwab@eng.au.dk (C.S.); 2 Department of Soil Science and Soil Protection, Czech University of Life Sciences Prague, Kamycka 129, 16500 Prague 6, Czech Republic; tejnecky@af.czu.cz

**Keywords:** amygdalin, arbutin, esculin, bifidobacteria, β-glucosidase, hydroquinone, antibacterial activity

## Abstract

Dietary plant glucosides are phytochemicals whose bioactivity and bioavailability can be modified by glucoside hydrolase activity of intestinal microbiota through the release of acylglycones. Bifidobacteria are gut commensals whose genomic potential indicates host-adaption as they possess a diverse set of glycosyl hydrolases giving access to a variety of dietary glycans. We hypothesized bifidobacteria with β-glucosidase activity could use plant glucosides as fermentation substrate and tested 115 strains assigned to eight different species and from different hosts for their potential to express β-glucosidases and ability to grow in the presence of esculin, amygdalin, and arbutin. Concurrently, the antibacterial activity of arbutin and its acylglycone hydroquinone was investigated. Beta-glucosidase activity of bifidobacteria was species specific and most prevalent in species occurring in human adults and animal hosts. Utilization and fermentation profiles of plant glucosides differed between strains and might provide a competitive benefit enabling the intestinal use of dietary plant glucosides as energy sources. Bifidobacterial β-glucosidase activity can increase the bioactivity of plant glucosides through the release of acylglycone.

## 1. Introduction

**Figure 1:**
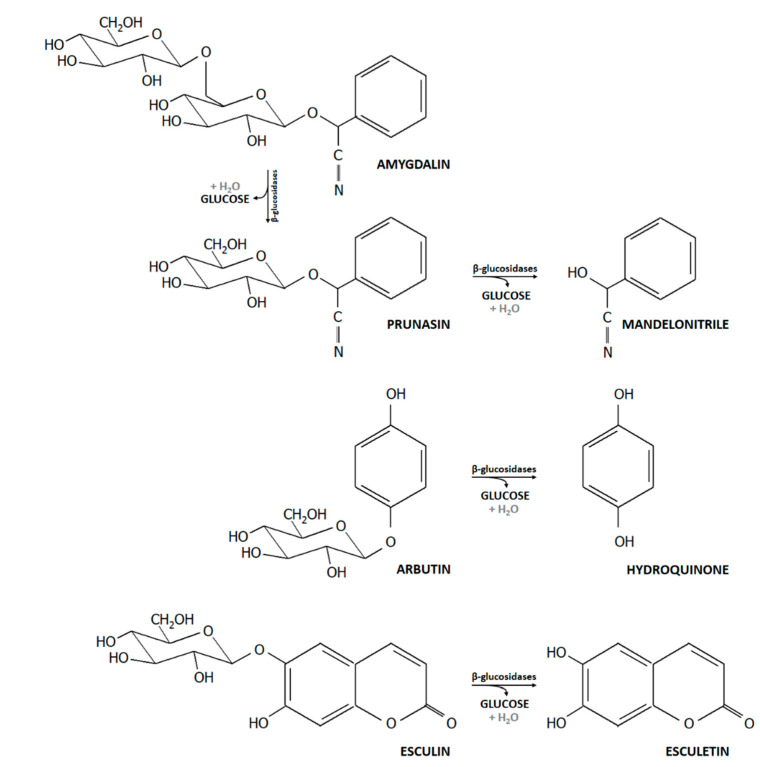
Structures of dietary plant glucosides (amygdalin, arbutin, and esculin) and their products after β-glucosidase hydrolysis.

Phytochemicals are found in leaves, fruits, vegetables, grains, and beans. Some glycosidic phytochemicals have been used in traditional medicine for centuries [1]. For example, the phenolic β-glucoside arbutin is a component of *Arctostaphylos uva-ursi* (bearberry leaf), which has been used in urinary tract infections [2]. Other plant derived β-glucosides include amygdalin (naturally occurring in almonds), esculin (dandelion coffee), fraxin (kiwi), polydatin (grapes), sinigrin (broccoli), and vanillin (vanilla) [3]. The biological effects of many glycosides are not attributed to their glycoside forms but to the corresponding aglycones (Figure 1). Aglycones are bioactive compounds that have lower molecular weight and hydrophilicity. After consumption, plant glucosides can be either taken up in the small intestine and undergo enterohepatic circulation, or they can be hydrolyzed by glycosidic activity of the gut microbiota [2]. Bacterial β-glucosidases, which have been classified within glycoside hydrolases (GH) families GH1, GH3, GH5, GH9, GH30, and GH116, cleave β-D-glucosidic linkages liberating glucose and the corresponding acylglycones. Some acylglycones have been shown to be antimicrobially active [4].

Several taxa of the major gut colonizers Firmicutes, Bacteroidetes and Actinobacteria possess β-glucosidase activity [5]. *Bifidobacterium* spp. (Actinobacteria) represent an important group of human commensals, being among the first microbial colonizers with considerable relevance for health in later life [6,7]. Intestinal competitiveness of bifidobacteria is attributed to their ability to degrade and metabolize a diversity of carbohydrates, and to carbohydrate resource sharing and cross-feeding [8,9]. Host adaptation seems to be linked to the ability to use dietary or host-derived glycans in a glycan-rich gut environment and differs between species and strains [10,11]. *Bifidobacterium* spp. frequently possess the potential to encode a wide array of glycosyl hydrolases, including β-glucosidases [12].

In humans, prevalence and diversity of members of the genus *Bifidobacterium* change in succession during life, with the species *Bifidobacterium longum* subsp. *infantis* and *Bifidobacterium bifidum* representing about 80% of the intestinal microbiota of infants, while *Bifidobacterium adolescentis*, *Bifidobacterium longum* subsp. *longum*, *Bifidobacterium breve*, *Bifidobacterium catenulatum*, and *Bifidobacterium pseudocatenulatum* prevail in adults representing about 1% of gut microbes [13,14,15,16,17,18]. Colonization of *B. bifidum* and *B. breve* seems to be limited to the human intestinal tract [12,19], whereas *B. adolescentis*, *B. longum* subsp. *longum*, *Bifidobacterium animalis* subsp. *animalis* and *lactis*, *B. catenulatum*, and *B. pseudocatenulatum* are considered multi-host species that were isolated from other mammals such as dogs, primates, and young ruminants on the milk diet [11,20]. *Bifidobacterium dentium* likely colonizes the oral cavity, and might only transiently pass the intestine [21,22].

*Bifidobacterium* spp. are common inhabitants of the human intestinal tract throughout life, and intestinal bifidobacterial β-glucosidase activity might modify the bioactivity and bioavailability of dietary plant glucosides. However, as there has been no systematic investigation, we tested 115 *Bifidobacterium* strains belonging to eight species that were associated with different hosts for β-glucosidase activity, the ability to grow in the presence of the coumarin glucoside esculin, the cyanogenic diglucoside amygdalin and the phenolic β-glucoside arbutin (Figure 1), and investigated the antibacterial activity of arbutin and its acylglycone hydroquinone. We additionally used genomic data of representative strains to screen for the presence of β-glucosidases encoding genes.

## 2. Materials and Methods

### 2.1. Bacterial Strains

Bifidobacterial strains (n = 115) of the species *B. adolescentis*, *B. animalis*, *B. bifidum*, *B. breve*, *B. dentium*, *B. longum*, and *B. catenutalum*, and *B. pseudocatenulatum* were obtained from the Deutsche Sammlung von Mikroorganismen und Zellkulturen (DSMZ, Germany) or the strain collection of the Department of Microbiology, Nutrition, and Dietetics (CZU, Czechia) (Table 1). Strain identity was confirmed with MALDI-TOF MS (Bruker Daltonik GmbH, Germany) according to Modrackova et al. (2019) [23]. MALDI-TOF MS failed to distinguish *B. catenulatum* and *B. pseudocatenulatum*, and to identify subspecies of *B. longum*. Subspecies of *B. animalis* were classified in previous studies [11,24,25]. Strains were routinely cultured in Wilkins–Chalgren broth (Oxoid, UK) supplemented with soya peptone (5 g L^−1^, Oxoid), L-cysteine (0.5 g L^−1^), and Tween 80 (1 mL L^−1^, both Sigma-Aldrich, USA) (WSP broth) in an oxygen-free carbon dioxide environment at 37 °C for 24 h. Stock cultures were stored at –80 °C in 30% glycerol and were reactivated in WSP broth for 24 h to obtain working cultures. Purity was routinely confirmed by phase-contrast microscopy.

### 2.2. Utilization of Selected Dietary Plant Glucosides

The ability of bifidobacteria to utilize glucosylated substrates was investigated in sterile 96-well microtiter plates (VWR, USA). Stock solutions of esculin (7-hydroxycoumarin-6-glucoside; Sigma-Aldrich), amygdalin (D-mandelonitrile-β-gentiobioside; Sigma-Aldrich), and arbutin (hydroquinone-β-D-glucopyranoside; Alfa Aesar, USA) were prepared in concentrations corresponding to 28 mM (5 g L^−1^) glucose (Penta, Czechia) in API 50 CHL medium (BioMérieux, France) with bromocresol purple as a pH indicator, and were filter sterilized. Glucose served as a growth positive control, API medium without added substrate was used to determine background growth.

Overnight cultures were centrifuged and re-suspended in the same volume of API medium. Strains were added (20 µL) to 180 µL API medium with or without added substrate. Plates were incubated under anaerobic conditions (GENbag anaer, bioMérieux, France) at 37 °C for 72 h. The color change from purple to yellow indicated a positive reaction. We measured absorbance at 434 nm and 588 nm using a Tecan Infinite M200 spectrometer (Tecan Group, Männedorf, Switzerland) and calculated the ration 434/588 [26], which was categorized as: no growth (<2); poor growth (2.1–2.4); growth (2.5–3.4); and very good growth (>3.5). Every strain was tested two or three times.

### 2.3. Metabolite Formation Analysis Using Ion Chromatography with Suppressed Conductivity Detection

Concentration of main fermentation metabolites lactate, acetate, and formate, was determined for selected strains using capillary high-pressure ion-exchange chromatography with suppressed conductivity detection. A Dionex ICS 4000 system equipped with IonPac AS11-HC 4 µm (Thermo Scientific, USA) guard and analytical columns. Eluent composition was as follows: 0–10 min isocratic: 1 mM KOH; 10–20 min linear gradient: 1–60 mM KOH; and 20–25 min again isocratic: 60 mM KOH. The flow rate was set to 0.012 mL min^−1^. An ACES 300 suppressor (Thermo Scientific, USA) was used to suppress eluent conductivity, while a carbonate Removal Device 200 (Thermo Scientific) was implemented to suppress carbon dioxide baseline shift. Chromatograms were processed with Chromeleon 7.20 (Dionex, USA). Standards were prepared from 1 g L^−1^ stock solutions (Analytika, Czechia; Inorganic Ventures, USA). Deionized water (conductivity <0.055 µS cm^−1^; Adrona, Latvia) was used for eluent and standard preparation (0.1–40 mg L^−1^).

### 2.4. Determination of Whole Cell β-Glucosidase Activity, and Release of the Acylglycone Esculetin

Beta-glucosidase activity of whole cells was assessed by enzymatic assay with 4-nitrophenyl β-D-glucopyranoside (PNP-G; Sigma-Aldrich, USA) as substrate. All samples were tested at least twice. Overnight cultures (1 mL) were centrifuged, supernatant was discarded and cell pellets were frozen at –20 °C. Frozen cells were re-suspended in 20 µL BifiBuffer (1.2 g L^−1^ K_2_HPO_4_, 0.333 g L^−1^ KH_2_PO_4_; Lachner, Czechia), 1 µL of this suspension was added to 99 µL of PNP-G solution (20 mM in Bifibuffer). Absorbance at 405 nm was measured before and after 4 h of incubation at 37 °C using a Tecan Infinite M200 spectrometer: reactions with a difference of absorbance >0.1 units were considered positive.

For selected strains, β-glucosidase activity was additionally tested using kits RAPID ID 32 A (bioMerieux, France), or ANAERO test 23 (Erba Lachema, Czechia) which employ PNP-G.

The release of esculetin from esculin was tested using ammonium iron citrate (Sigma Aldrich, USA) as scavenger, the reaction of esculetin with ferric ions changes the color from purple to opaque black. Bifidobacteria were inoculated in API medium supplied with 10.2 g L^−1^ esculin (corresponding to 28 mM solution of glucose) and 1 g L^−1^ ammonium iron citrate in microtiter plates as described above; and were incubated under anaerobic conditions (GENbag anaer) at 37 °C for 72 h. The color change was assessed visually. Every strain was tested at least twice.

### 2.5. Antibacterial Activity of Arbutin and Hydroquinone against Selected Bifidobacterium Strains

The antibacterial activity of arbutin and its acylglycone hydroquinone (Sigma-Aldrich, St. Louis, MI, USA) was tested using two-fold broth dilution assay in 96-well sterile microtiter plates. Overnight cultures of selected strains (DSM 20083, DSM 20104, DSM 10140, DSM 20456, DSM 20213, DSM 20211, DSM 20219, and DSM 20088), which represented five of the species tested (Table 2), grown in WSP broth, were centrifuged, and the cell pellet was resuspended in the same volume of API medium supplied with 14 mM glucose. A two-fold dilution series was prepared in microtiter plates using API stock solutions containing 14 mM glucose, and 28 mM arbutin or hydroquinone. Cultures (10%) were added, and plates were incubated under anaerobic conditions (GENbag anaer) at 37 °C for 24 h. Absorbance at 434 and 588 nm was determined using a spectrophotometer and the ratio of 434 nm/588 nm was calculated as described above. The minimal inhibitory concentration (MIC) was defined as the concentration that prevented growth, metabolite formation and thereby color change of the API medium. Every strain was analyzed at least three times.

To test whether the presence of arbutin in API medium impacted growth, we additionally conducted growth kinetics of selected strains that were able or lacked the ability to grow with arbutin in API medium supplied with 28 mM glucose, 14 mM glucose and 14 mM arbutin, or 28 mM arbutin to ensure the availability of the same concentration of glucose. Strains were grown in microtiter plates as described before, and absorbance was measured at 0, 3, 6, 9, 12, 24, 30, 36, and 48 h at 434 and 588 nm, to calculate the ratio of 434 nm/588 nm as described above.

### 2.6. Identification and Comparison of β-Glucosidases Encoded by Representative Bifidobacterium spp. of the Species Investigated

Homologues of previously characterized β-glucosidases of *B. animalis* subsp. *lactis* [27] and *B. pseudocatenulatum* IPLA 36007 [28] were identified in genomes of *B. adolescentis* DSM 20083 (AP009256), *B. animalis* subsp. *lactis* BB12 (CP001853.1), DSM 10140 (CP001606.1), *B. animalis* subsp. *animalis* DSM 20104 (CP002567.1), *B. bifidum* DSM 20456 (AP012323.1), *B. breve* DSM 20213 (ACCG02000000), *B. longum* subsp. *longum* DSM 20219 (AP010888.1), *B. longum* subsp. *infantis* DSM 20088 (CP001095.1), *B. longum* subsp. *suis* 20211 (JGZA01000002.1), *B. catenulatum* DSM 16992 (ABXY01000009.1), *B. catenulatum* subsp. *kashiwanohense* DSM 21854 (JGYY01000015.1), *B. pseudocatenulatum* DSM 20438 (ABXX02000004.1) and *B. dentium* DSM 20436 (FNSE01000001.1) using blastP. To identify additional β-glucosidases, genomic data were obtained from NCBI and were annotated with RAST using default settings [29]. Glycosyl hydrolases of family 1 and 3 were identified using the dbCAN database based on a search for signature domains of every CAZyme family [30].

## 3. Results

### 3.1. Distribution of (Putative) β-Glucosidases Encoding Genes in Genomes of Representative Bifidobacterium spp.

We screened the genomes of selected strains for the presence of GH1 and GH3 encoding genes (Table 3). Homologous proteins related to the four GH3 β-glucosidases characterized in *B. pseudocatenulatum* IPLA 36007 were present in *B. adolescentis* DSM 20083, *B. breve* DSM 20213, *B. catenulatum* subsp. *kashiwanohense* DSM 21854, *B. catenulatum* DSM 16992, and *B. pseudocatenulatum* DSM 20438. Multiple β-glucosidases (n = 1 GH1, and n = 11 GH3) were encoded by the genome of *B. dentium* DSM 20438 including homologues to the four β-glucosidases of *B. pseudocatenulatum* IPLA 36007. Strains of *B. animalis* harbored a homologue of Bbg572 (GH1) of *B. animalis* subsp. *lactis* SH5, and in addition a homologue of r-β-gluE of *B. pseudocatenulatum* IPLA 36007. The distribution of β-glucosidases in *B. longum* differed between subspecies, *B. longum* subsp. *longum* DSM 20219 and *B. longum* subsp. *suis* DSM 20211 possessed homologues of r-β-gluE, which were also highly similar (>96%) to a characterized β-glucosidase of *B. longum* H1 [31], while r-β-gluD was present in all three subspecies but was truncated in *B. longum* subsp. *longum* DSM 20219. *B. longum* subsp. *infantis* DSM 20288 additionally possessed a homologue of Bbg572. *B. bifidum* DSM 20456 harbored only one putative GH1 β-glucosidase with low homology to Bbg572.

### 3.2. Beta-Glucosidase Activity of Bifidobacterium spp.

Whole cell β-glucosidase activity was investigated by enzymatic assay using PNP-G as substrate. *B. adolescentis*, *B. animalis*, *B. breve*, *B. catenulatum/pseudocatenulatum*, and *B. dentium* were consistently β-glucosidase positive (Table 1). In contrast, all tested strains of *B. bifidum*, with a few exceptions, and most strains of *B. longum,* were β-glucosidase negative. From *B. longum*, only four strains that originated from dog feces (10/6b, 32/3na, 33/5nb, and 33/4nc), two isolates from stool of adults (B1, PEG080) and one from pig feces (DSM 20211) showed β-glucosidase activity. For selected strains, β-glucosidase activity was confirmed using RAPID ID 32 A and ANAEROtest 23 (Table 1).

### 3.3. Growth in the Presence of β-Glucosides

We observed that β-glucosidase activity is a common yet species dependent feature of *Bifidobacterium* spp. To investigate whether β-glucosidase activity relates to the ability to use plant glucosides, we grew strains with esculin, amygdalin, and arbutin as a sole carbohydrate source in API 50 CHL medium (Table 1). All strains were able to grow in the presence of glucose, verifying the suitability of the assay.

In general, amygdalin was the preferred β-glucosylated substrate used by 54% of strains, followed by esculin (47%), and arbutin (24%).

Strains belonging to *B. dentium* were most versatile in the utilization of the provided β-glucosides as all strains grew in the presence of amygdalin and esculin. Only three *B. dentium* strains (DSM 20436, TH1, and VOK II) were not able to use arbutin. All strains of *B. breve* used amygdalin and esculin (with one exception, B43), while the utilization of arbutin was less frequent (46%). Within *B. catenulatum/pseudocatenulatum*, 83% strains grew in the presence of amygdalin, while 67% utilized esculin and 38% arbutin. The majority of the *B. adolescentis* strains (69%) was capable of using amygdalin, strains B35 and B39 grew in presence of arbutin and esculin. For *B. animalis*, we observed subspecies dependent differences in substrate utilization. The majority of *B. animalis* subsp. *lactis* strains, except three isolates from dog feces (P2N1, 43/7nb, and 11/6a), utilized amygdalin (80%), 67% used esculin, and only PEG084 grew in the presence of arbutin. In contrast, *B. animalis* subsp. *animalis* strains were not capable to grow with amygdalin and arbutin, while 57% utilized esculin. None of the *B. longum* strains was able to use esculin and arbutin, amygdalin utilization was detected for *B. longum* DSM 20211 and PEG080, while *B. bifidum* strains were not able to utilize any of the provided plant β-glucosides.

### 3.4. Release of the Acylglycone Esculetin

We qualitatively determined whether β-glucoside hydrolysis of esculin would lead to the release of the acylglycone esculetin using ammonium iron citrate as a scavenger (Table 1). The majority (94%) of the strains that were positive in the PNP-G enzymatic assay released esculetin confirming β-glucosidase activity. Few strains (namely DSM 20211, B1, 32/3na, 33/5nb, and 33/4nc), all from *B. longum*, were PNP-G positive, while esculetin release was not detected. More than half (66%) of the strains that were able to release esculetin grew when esculin was present as substrate.

### 3.5. Metabolite Formation during Growth of Bifidobacteria in the Presence of Plant Glucosides

**Figure 2:**
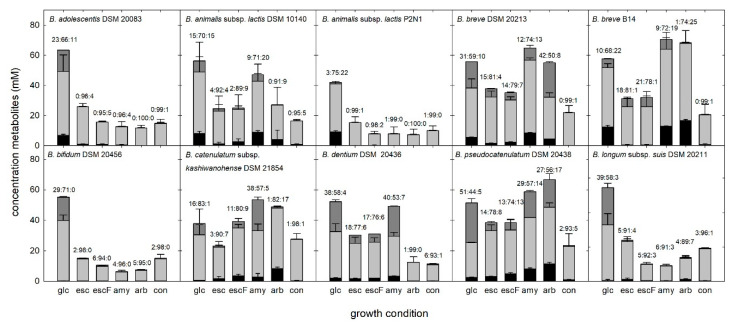
Metabolites formed during growth in the presence of glucose or plant glucosides. Formation of fermentation metabolites, lactate (dark grey), acetate (light grey), and formate (black), by selected strains grown in API 50 CHL medium supplied with plant glucosides (equivalent to 28 mM glucose) was analyzed after 72 h incubation by capillary ion-exchange chromatography with suppressed conductivity. The proportion of major metabolites lactate, acetate, and formate for each growth condition are shown above the bar. Strains were tested two or three times. glc, glucose; esc, esculin; escF, esculin and ammonium iron citrate; amy, amygdalin; arb, arbutin; and con, negative control.

For representative strains from different species, we investigated metabolite formation as an indicator of fermentation activity. Lactate, acetate, and formate concentrations were measured by capillary ion-exchange chromatography with suppressed conductivity detection (Figure 2). All strains grew in the presence of glucose, producing mainly acetate metabolite (44%–83% of metabolites formed), followed by lactate (3%–51%) and formate (0%–22%). In negative controls (API medium without added glycan source), and samples without visible growth, acetate was formed as the major metabolite, otherwise the acetate:lactate:formate ratios differed between strains, and compared to growth in glucose.

### 3.6. Antibacterial Activity of Hydroquinone and Arbutin and Impact on Growth Kinetics

We tested the antibacterial and growth affecting activity of arbutin and its acylglycone hydroquinone on representative strains using two-fold dilution assay in microtiter plates and growth kinetics, respectively. Glucose (14 mM) was added to API medium to avoid growth inhibition due to substrate limitation. None of the strains were affected by the presence of arbutin even at the maximum concentration tested (25.5 mM) (Table 2). *B. animalis* subsp. *animalis* DSM 20104, *B. longum* subsp. *longum* DSM 20019, *B. longum* subsp. *suis* DSM 20211, and *B. longum* subsp. *infantis* DSM 20088 were most sensitive towards hydroquinone with MIC ≤ 0.05 mM while the MIC of the other strains was 0.1–0.2 mM (Table 2).

**Figure 3:**
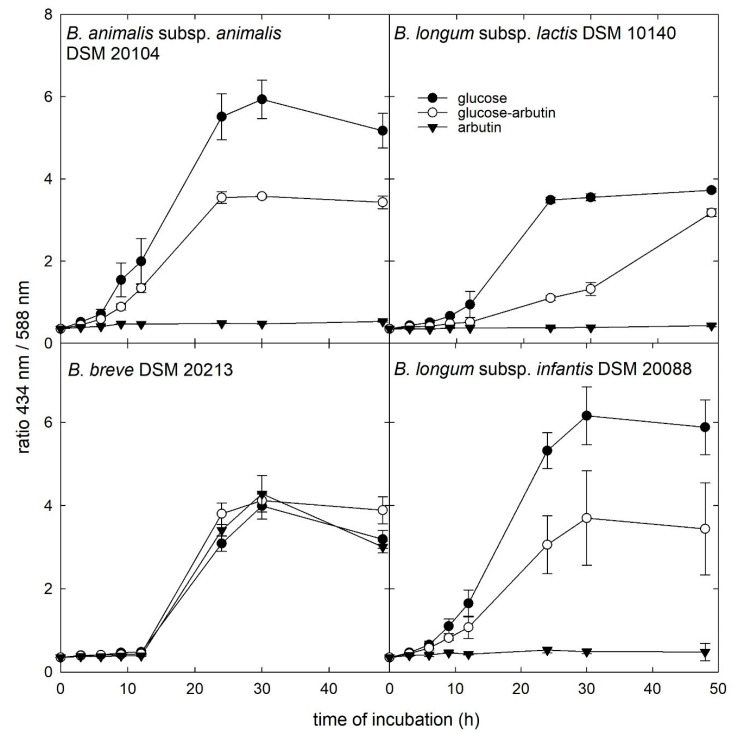
Impact of arbutin addition on growth. Growth kinetics of selected strains were tested in microtiter plates with API medium supplied with 28 mM glucose, 14 mM glucose and arbutin, or 28 mM arbutin. The plates were spectrophotometrically measured at 0, 3, 6, 9, 12, 24, 30, 36, and 48 h at 434 and 588 nm, to calculate the ratio of 434 nm/588 nm.

Strains *B. animalis* subsp. *animalis* DSM 20104 and *B. animalis* subsp. *lactis* DSM 10140, *B. breve* DSM 20213, and *B. longum* subsp. *infantis* DSM 20088 were additionally grown in the presence of glucose, glucose and arbutin, or arbutin (Figure 3). The presence of arbutin did not impact the growth of *B. breve* DSM 20213, while the lag phase of the other strains was delayed in the presence of glucose and arbutin and the final absorbance ratio reached was approximately 50% compared to glucose only.

## 4. Discussion

Plant derived β-glucosides encompass structurally diverse compounds, which, when ingested, can reach the colon and be enzymatically modified by gut microbes. Beta-glucosidases are widely distributed in gut microbes and play important roles in biological processes. Here, we demonstrate species and strain dependent variability of *Bifidobacterium* spp. in the utilization of dietary plant glucosides linked to aromatic aglycones.

### 4.1. Genomic Potential for β-Glucosidase Activity Partly Predicts Activity

Based on genomic data, strains of the phylogenetically closely related species *B. adolescentis*, *B. catenulatum*, *B. pseudocatenulatum*, *B. dentium*, and the more distant *B. breve* [10], harbored a core of four β-glucosidases. In agreement, all strains of these species possessed β-glucosidase activity but showed preference for different substrates. Indeed, the purified β-glucosidases of *B. pseudocatenulatum* IPLA 36007 varied in their ability to release of the aglycones daidzein and genistein from isoflavone glycosides daidzin and genistin, indicating enzyme dependent substrate preference [28].

Strains of *B. longum* did not show β-glucosidase activity despite the presence of genes encoding β-glucosidases with high homology to a purified β-glucosidases of *B. longum* H1, which hydrolyzed arbutin. Lack of β-glucosidase activity of whole cells might suggest that enzymes were either not expressed or expressed at concentrations not sufficient to confer activity at test conditions.

*B. animalis* subsp. *lactis* and *B. animalis* subsp. *animalis* harbored highly similar GH1 and GH3 β-glucosidases and possessed β-glucosidase activity. The similar GH1 β-glucosidase Bgl572 hydrolyzed PNP-G and arbutin when purified [31]. However, growth in the presence of plant glucosides differed between *B. animalis* subspecies, possibly due different transport mechanism or sensitive towards the released acylglycones. Indeed, *B. animalis* subsp. *animalis* DSM 20104 was more sensitive towards hydroquinone than *B. animalis* subsp. *lactis* DSM 10140.

### 4.2. Bifidobacterium β-Glucosidase Increases Bioactivity and Bioavailability of Plant Glucosidases and Acylglycones

Plants are used for antibacterial properties in medical applications, and bacterial β-glucosidase activity leads to the release of bioactive acylglycones. Indeed, we confirmed the release of esculetin from esculin, by almost all strains with β-glucosidase activity, modifying bioactivity and bioavailability. Despite the ability to hydrolyze esculin, not all strains were able to grow when esculin was supplied as substrate, which might be due to the antimicrobial activity of esculetin [33].

Bioactivity of plant glucosides is likely lower than of acylglycones due to larger molecular weight and higher hydrophobicity. In confirmation, arbutin at the concentrations tested did not show inhibition while hydroquinone conferred strong antibacterial activity against the *Bifidobacterium* strains tested. Hydroquinone MIC values of bifidobacteria ranging from ≤0.05–0.2 mM were lower than reported for *Staphylococcus aureus* (1–11 mM) [34,35] and various aerobic Gram-positive and -negative strains [4] including *Enterococcus faecalis*, *Escherichia coli*, *Pseudomonas aeruginosa*, and *Klebsiella pneumonia* (1.5–6 mM).

MIC were up to 10-fold lower than the levels of hydroquinone theoretically released during growth. However, in the MIC assay, a low concentration of bacterial cells is exposed to high concentrations of the antibacterial at the beginning of the growth phase, while in the growth assay, increasing amounts of hydroquinone face an increasing number of bacterial cells. Sensitivity of bifidobacteria to hydroquinone, which, if released in the intestinal tract, could reduce the growth potential of β-glucosidase active strains, but also of neighboring cells.

In addition, β-glucosidase activity of *Bifidobacterium* spp. increased the bioavailability of acylglycones. Hydroquinone has been linked to anticancer activity in vitro and in vivo [36] and has been shown to confer antimycobacterial and antileishmanial activity in vitro [37].

### 4.3. Niche Adaption of Bifidobacteria Seems Related to β-Glucosidase Activity

Host adaptation of *Bifidobacterium* spp. was suggested to be linked to the ability to use dietary or host-derived glycans and differs between species and strains [10,11]. Indeed, strains of *B. bifidum* and *B. longum* subsp. *infantis*, which are among the most abundant microbes during the first months of life, lacked β-glucosidase activity in agreement to previous observations [5], and with the absence of β-glucosidase encoding genes in the genomes. Both species occur in the infant gut when glycan substrates are limited to human breast milk, endogenous mucin, or infant formula. Indeed, a previous cohort of studies observed that fecal β-glucosidase activity was low after birth and gradually increased with the introduction of a more diverse diet [38]. Interestingly, *B. animalis* subsp. *animalis* showed only little growth in the presence of plant glucosides despite possessing β-glucosidase activity. Most isolates were obtained from lamb and calf feces early when animals received a milk diet.

*B. breve* colonizes infant and adults, and similar to *B. adolescentis* and other adult or multi-host species, possesses multiple β-glucosidases. In the adult gut, *Bifidobacterium* spp. contribute a minor share of the population, and compete with other β-glucosidase positive taxa for substrate [5]. Beta-glucosidase activity of *Bifidobacterium* strains colonizing adults might enhance ecological competitiveness.

For the multi-host subspecies of *B. animalis* subsp. *lactis*, plant glucoside utilization profiles likewise suggested host adaption in agreement with genetic and phenotypic host-specific differences previously observed [11]. Strains from adult ruminant hosts (Cameroon sheep, Barbary sheep, and an okapi), that naturally receive a plant-based diet, used plant glucosides in contrast to strains isolated from omnivorous dogs.

### 4.4. Substrate Source Impacted Fermentation Profiles

Bifidobacteria metabolize hexoses via the “bifid shunt” with fructose-6-phosphoketolase as the key enzyme. Glucose (1 mol) theoretically yields 1.5 mol acetate, 1 mol lactate, and 2.5 ATP [39]. Whether the intermediate pyruvate is cleaved to acetyl phosphate and formate, or reduced to lactate, depends on type and supply of the substrate carbohydrate [40], possibly in the presence of oxygen [8] and on different rates in substrate consumption. A previous study reported that with a decrease of consumption rate, relatively more acetic and formic acid and less lactic acid was produced by *Bifidobacterium* spp. [41]. Indeed, the proportion of lactate was reduced for most strains grown in the presence of esculetin, and to a lesser extent with arbutin, which might indicate that hydrolysis activity reduced the consumption rate.

## 5. Conclusions

Our data shows that bifidobacterial β-glucosidase activity is preserved among species which might be related to niche adaption. Structural homology of a core set of β-glucosidases of species associated with adult humans could suggest that these enzymes evolved together. Beta-glucosidase activity may provide a competitive advantage in the mammalian gut proving access to energy sources, but they might also have environmental impact due to the release of bioactive antibacterial acylglycones; thus, increasing bioavailability due to the formation of fermentation metabolites.

## Figures and Tables

**Table 1:** Bifidobacterial utilization of β-glucosylated substrates during growth, and β-glucosidase activity. The ability to utilize plant glucosides was tested in API 50 CHL media. The color change detection of cultivation media after 72 h of incubation at 37 °C under anaerobic conditions was determined spectrophotometrically (434 nm/588 nm). A ratio of <2 was considered no growth (-); 2.1–2.4: poor growth (+); 2.5–3.4: growth (++); and >3.5: very good growth (+++). Beta-glucosidase activity was tested by enzymatic assay with 4-nitrophenyl β-D-glucopyranoside (β-GLU) as substrate with spectrophotometric measurement at 405 nm; the difference of >0.1 was considered positive (+). The release of the acylglycone from esculin was determined visually using ammonium iron citrate as scavenger (ESN rel.).

Species/Subspecies	Strain	Origin	GLU	ESC	AMYG	ARB	API	β-GLU	ESN rel.
*B. adolescentis*	DSM 20083	Intestine of adult	+++	-	-	-	-	+	+
	B34	Stool of infant	+++	-	+++	-	-	+	+
	B35	Stool of infant	+++	++	+++	+	-	+	+
	B36	Stool of infant	+++	-	-	-	-	+^R^	+
	B38	Stool of infant	+++	+	-	-	-	+	+
	B2	Stool of adult	++	-	++	-	-	+^R^	+
	B9	Stool of adult	++	-	++	-	-	+^R^	+
	B30	Stool of adult	+++	-	+++	-	-	+^R^	+
	B39	Stool of adult	+++	++	+++	+++	-	+	+
	B41	Stool of adult	+++	-	+++	-	-	+	+
	B56	Stool of adult	++	-	-	-	-	+	+
	PEG038	Stool of adult	+++	-	+++	-	-	+	+
	10/6d	Dog feces	+++	-	+++	-	-	+	+
*B. animalis* subsp. *animalis*	DSM 20104	Rat feces	+++	-	-	-	-	+	+
	805P4	Calf feces	+++	+++	-	-	-	+^R^	+
	012II1	Calf feces	+++	+	-	-	-	+^R^	+
	023II	Calf feces	+++	-	-	-	-	+^R^	+
	J1 (L1)	Lamb feces	++	-	-	-	-	+^R^	+
	J5 (L4)	Lamb feces	+++	+	-	-	-	+^R^	+
	J6 (L3)	Lamb feces	+++	++	-	-	-	+	+
*B. animalis* subsp. *lactis*	DSM 10140	Yoghurt	+++	-	+++	-	-	+	+
	BB12	Probiotic product	++	++	++	-	-	+	+
	Dan	Probiotic product	++	+	+++	-	-	+	+
	Nestlé	Infant nutrition	+++	++	++	-	-	+	+
	S7	Ovine cheese	++	++	++	-	-	+	+
	B22	Stool of infant	+++	++	+++	-	-	+	+
	B25	Stool of infant	+++	+	+++	-	-	+	+
	PEG042	Stool of adult	+++	-	+++	-	-	+	+
	PEG084	Stool of adult	++	+	++	+++	-	+	+
	P2N1	Dog feces	+++	-	-	-	-	+	+
	43/7nb	Dog feces	+++	-	-	-	-	+	+
	11/6a	Dog feces	++	-	-	-	-	+	+
	ZDK1	Cameroon sheep feces	+++	++	+++	-	-	+	+
	ZDK4	Barbary sheep feces	++	++	++	-	-	+	+
	ZDK7	Okapi feces	+++	++	+++	-	-	+	+
*B. bifidum*	DSM 20456	Stool of infant	+++	-	-	-	-	-	-
	DSM 20239	Stool of infant	+++	-	-	-	-	-	-
	B6	Stool of infant	+++	-	-	-	-	-^R^	-
	B33	Stool of infant	+++	-	-	-	-	-^R^	-
	B10	Stool of adult	+++	-	-	-	-	-^R^	-
	B29	Stool of adult	+++	-	-	-	-	-^R^	-
	B40	Stool of adult	+++	-	-	-	-	-	-
	B55	Stool of adult	+++	-	-	-	-	-	-
*B. breve*	DSM 20213	Intestine of infant	+++	++	+++	+++	-	+	+
	BR03	Probiotic product	++	++	+++	-	-	+	+
	B13	Stool of infant	+++	+++	+++	++	-	+^R^	+
	B14	Stool of infant	+++	++	+++	+++	-	+^R^	+
	B37	Stool of infant	+++	+++	+++	-	-	+	+
	B42	Stool of infant	++	++	++	+++	-	+	+
	B43	Stool of infant	+++	-	++	-	-	+	+
	B50	Stool of infant	+++	+++	+++	+++	-	+	+
	B57	Stool of infant	+++	+++	+++	-	-	+	+
	PEG010	Stool of adult	++	++	+++	-	-	+	+
	PEG064	Stool of adult	+++	+++	+++	-	-	+	+
	PEG071	Stool of adult	+++	++	+++	-	-	+	+
	PEG074	Stool of adult	+++	+++	+++	++	-	+	+
*B. catenulatum* *B. catenulatum* subsp. *kashiwanohense* *B. pseudocatenulatum* *B. catenulatum/* *pseudocatenulatum*	DSM 16992	Human feces	+++	+++	+++	+++	-	+	+
	DSM 21854	Stool of infant	+++	-	+++	+	-	+	+
	DSM 20438	Stool of infant	+++	+++	+++	+++	-	+	+
	B12	Stool of infant	+++	-	+++	-	-	+^R^	+
	B46	Stool of infant	+++	++	+++	+++	-	+	+
	B48	Stool of infant	+++	+++	-	-	-	+	+
	B23	Stool of adult	+++	++	+++	-	-	+^R^	+
	B32	Stool of adult	+++	++	+++	-	-	+^R^	+
	B51	Stool of adult	++	-	+++	-	-	+	+
	B52	Stool of adult	+++	+	-	+++	-	+	+
	B53	Stool of adult	+++	-	+++	-	-	+	+
	22/4nb	Dog feces	+++	++	+++	-	-	+	+
*B. dentium*	DSM 20436	Dental caries	+++	+++	+++	-	-	+	+
	FD1	Stool of infant	+++	++	+++	+++	-	+ ^A^	+
	TH1	Stool of infant	+++	+++	+++	-	-	+ ^A^	+
	VOK II	Stool of infant	+++	++	+++	-	-	+ ^A^	+
	PEG020	Stool of adult	+++	++	+++	+++	-	+	+
	A1/5A	Monkey feces	+++	+++	+++	+++	-	+ ^A^	+
	N12	Monkey feces	+++	+++	+++	+++	-	+ ^A^	+
	N21	Monkey feces	+++	+++	+++	+++	-	+ ^A^	+
	N23	Monkey feces	+++	+++	+++	+++	-	+ ^A^	+
	N26	Monkey feces	+++	+++	+++	+++	-	+ ^A^	+
	N77	Monkey feces	+++	+++	+++	+++	-	+ ^A^	+
	N79	Monkey feces	+++	+++	+++	+++	-	+ ^A^	+
	N105	Monkey feces	+++	+++	+++	+++	-	+ ^A^	+
	N109	Monkey feces	+++	+++	+++	+++	-	+ ^A^	+
	N110	Monkey feces	+++	+++	+++	+++	-	+ ^A^	+
	N111	Monkey feces	+++	+++	+++	+++	-	+ ^A^	+
	N112	Monkey feces	+++	+++	+++	+++	-	+ ^A^	+
*B. longum* subsp. *infantis* *B. longum* subsp. *longum* *B. longum* subsp. *suillum* *B. longum* subsp. *suis* *B. longum*	DSM 20088	Stool of infant	+++	-	-	-	-	-	-
	DSM 20219	Intestine of adult	+++	-	-	-	-	-	-
	DSM 28597	Feces of piglets	+++	-	-	-	-	-^A^	-
	DSM 20211	Pig feces	+++	-	+++	-	-	+	-
	5/9	Calf feces	+++	-	-	-	-	-	-
	INFNUT	Probiotic product	++	-	-	-	-	-	-
	B3	Stool of infant	+++	-	-	-	-	-^R^	-
	B4	Stool of infant	++	-	-	-	-	-^R^	-
	B7	Stool of infant	+++	-	-	-	-	-	-
	B8	Stool of infant	+++	-	-	-	-	-	-
	B11	Stool of infant	+++	-	-	-	-	-	-
	B16	Stool of infant	+++	-	-	-	-	-	-
	B17	Stool of infant	+++	-	-	-	-	- ^R^	-
	B19	Stool of infant	+++	-	-	-	-	-^R^	-
	B20	Stool of infant	++	-	-	-	-	-^R^	-
	B27	Stool of infant	++	-	-	-	-	-	-
	B28	Stool of infant	+++	-	-	-	-	-	-
	B44	Stool of infant	+++	-	-	-	-	-^A^	-
	B49	Stool of infant	+++	-	-	-	-	-	-
	B1	Stool of adult	+++	-	-	-	-	+	-
	B26	Stool of adult	+++	-	-	-	-	-^R^	-
	PEG057	Stool of adult	+++	-	-	-	-	-	-
	PEG059	Stool of adult	+++	-	-	-	-	-	-
	PEG080	Stool of adult	++	-	++	-	-	+	+
	PEG104	Stool of adult	+++	-	-	-	-	-	-
	022II	Calf feces	+++	-	-	-	-	-^R^	-
	10/6b	Dog feces	+++	-	-	-	-	+	+
	32/3na	Dog feces	+++	-	-	-	-	+	-
	33/5nb	Dog feces	++	-	-	-	-	+	-
	33/4nc	Dog feces	++	-	-	-	-	+	-

GLU, glucose; ESC, esculin; AMYG, amygdalin; ARB, arbutin; API, negative control; β-GLU, β-glucosidase activity; ESN rel., esculetin release; superscript letter A, the shown reaction (positive/negative) of β-glucosidase activity is confirmed by ANAEROtest 23; and superscript letter R, the shown reaction of β-glucosidase activity is confirmed by RAPID ID 32 A.

**Table 2:** Minimal inhibitory concentrations of hydroquinone and arbutin. Minimal inhibitory concentrations (MIC) were determined using a two-fold dilution assay in microtiter plates and API medium supplied with glucose (14 mM), and hydroquinone or arbutin. The MIC was defined as the concentration that completely inhibited growth of strains determined using the absorbance ration 434 nm/588 nm. MIC were tested in 3–5 independent replicates.

Species or Subspecies	Strain	Minimal Inhibitory Concentration (mM)	
		Hydroquinone	Arbutin
*B. adolescentis*	DSM 20083	0.05–0.10	>25.5
*B. animalis* subsp. *animalis*	DSM 20104	≤0.05	>25.5
*B. animalis* subsp. *lactis*	DSM 10140	0.10–0.20	>25.5
*B. bifidum*	DSM 20456	0.10–0.20	>25.5
*B. breve*	DSM 20213	0.10–0.20	>25.5
*B. longum* subsp. *suis*	DSM 20211	≤0.05	>25.5
*B. longum* subsp. *longum*	DSM 20219	≤0.05	>25.5
*B. longum* subsp. *infantis*	DSM 20088	≤0.05	>25.5

**Table 3:** Distribution of β-glucosidases in representative strains of *Bifidobacterium* species. Beta-glucosidases putatively encoded by the genomes were compared to characterized β-glucosidases of *B. animalis* subsp. *lactis* SH5 (Bbg572, GH1) or to four GH3 β-glucosidases r-β-gluA, r-β-gluB, r-β-gluD, and r-β-gluE of *B. pseudocatenulatum* IPLA36007.

Species	Strain	Characterized Beta-Glucosidase					Not Yet Characterized β-Glucosidases
		GH Family 1	GH family 3				
		Bbg572 (JX274651) 461 AA	r-β-gluE (AW18_08090, KEF28001.1) 787 AA	r-β-gluB (AW18_09810, KEF27912.1) 809 AA	r-β-gluD (AW18_08145, KEF28010.1) 748 AA	r-β-gluA (AW18_01575, KEF29323.1) 964 AA	
*B. breve*	DSM 20213	-	EFE88733.1A 93% I, 90% P in 774 AA	EFE90113.1 80% I, 97% P in 833 AA	EFE88739.1 82% I, 90% P in 757 AA	EFE90117.1 70% I, 81% P in 811 AA	
*B. adolescentis*	DSM 20083	-	BAF39978.1 96% I, 98% P in 780 AA	BAF40379.1 90% I, 94% P in 811 AA	BAF39975.1 ^B^ 85% I, 92% P in 748 AA	BAF40392.1 97% I, 93% P in 962 AA	BAF40391.1
*B. longum* subsp. *longum*	DSM 20019	-	BAJ67169.1 ^C^ 82% I, 89% P in 776 AA	-	BAJ67164.1 78% I, 87% P in 507 AA ^D^	-	
*B. longum* subsp. *suis*	DSM 20211	-	KFI73778.1 ^E^ 82% I, 90% P in 775 AA	-	KFI73782.1 83% I, 91% P in 752 AA	-	KFI73422.1
*B. longum* subsp. *infantis*	DSM 20088	ACJ52977.1 69% I, 81% P in 417 AA	-	-	ACJ51732.1 82% I, 90% P in 756 AA	-	
*B. animalis* subsp. *animalis*	DSM 20104	AFI62379.1 96% I, 98% P in 460 AA	AFI63691.1 73% I, 84% P in 776 AA	-	-	-	
*B. animalis* subsp. *lactis*	DSM 10140	ACS47112.1 100% I, 100% P in 476 AA	ACS48458.1 73% I, 84% P in 771 AA	-	-	-	
*B. animalis* subsp. *lactis*	BB12	ADC85172.1 100% I, 100% P in 460 AA	ADC84934.1 73% I, 84% P in 771 AA	-	-	-	ADC84934.1
*B. bifidum*	DSM 20456	BAQ97280.1 47% I, 63% P in 437 AA	-	-	-	-	
*B. catenulatum* subsp. *kashiwanohense*	DSM 21854	KFI63440.1 71% I, 82% P in 458 AA	KFI67404.1 97% I, 98% P in 780 AA	KFI63941.1 95% I, 97% P in 728 AA	KFI67400.1 98% I, 99% P in 748 AA	KFI63834.1 92% I, 99% P in 299 AA*	
*B. catenulatum*	DSM 16992	-	EEB22212.1 96% I, 98% P in 780 AA	EEB21148.1 95% I, 97% P in 809 AA	EEB22216.1 98% I, 99% P in 748 AA	EEB22373.1 93% I, 96% P in 696 AA	EEB22212.1
*B. pseudocatenulatum*	DSM 20438	-	EEG71163.1 96% I, 98% P in 780 AA	EEG71238.1 99% I, 99% P in 809 AA	EEG71159.1 98% I, 99% P in 748 AA	EEG70226.1 99% I, 99% P in 964 AA	
*B. dentium*	DSM 20436	SEC02936.1 69% I, 80% P in 457 AA	SEC47920.1 99% I, 95% P in 774 AA	SEC11609.1 87% I, 93% P in 809 AA	SEC48734.1 94% I, 98% P in 748 AA	SEC11364.1 90% I, 95% P in 962 AA SEC11543.1 61% I, 76% P in 962 AA	SEC18208.1 SEB97687.1 SEB79266.1 SEC47658.1 SEC14043.1 SEB96905.1

^A^ I = Identities, P = Positives; ^B^ BaBgl3 was characterized by Florindo et al. (2018) [32]; ^C^ 96% I, 98% P in 783 AA to β-glucosidase of *B. longum* H1 [31]; ^D^ truncated protein; ^E^ 96% I, 98% P in 787 AA to β-glucosidase of *B. longum* H1 [31].
